# Medicine donation programmes supporting the global drive to end the burden of neglected tropical diseases

**DOI:** 10.1093/trstmh/traa167

**Published:** 2021-01-16

**Authors:** Mark Bradley, Rachel Taylor, Julie Jacobson, Morgane Guex, Adrian Hopkins, Julie Jensen, Lynn Leonard, Johannes Waltz, Luc Kuykens, Papa Salif Sow, Ulrich-Dietmar Madeja, Takayuki Hida, Kileken Ole-Moiyoi, Jonathan King, Daniel Argaw, Jamsheed Mohamed, Maria Rebollo Polo, Aya Yajima, Eric Ottesen

**Affiliations:** Global Health, GSK, Brentford, UK; Corporate Responsibility, Merck & Co., Inc., Kenilworth, NJ, USA; Bridges to Development, Seattle, Washington, USA; International Federation of Pharmaceutical Manufacturers and Associations, Geneva, Switzerland; Adrian Hopkins Consulting, Gravesend, Kent, UK; Global Health & Patient Access, Pfizer, Inc. New York, NY, USA; Johnson & Johnson Global Public Health, New Brunswick, NJ, USA; Global Health, Group Corporate Affairs Merck KGaA, Darmstadt, Germany; Global Health Programs, Sanofi, Paris, France; Global Patient Solutions Africa, Gilead Sciences, Foster City, CA, USA; Neglected Tropical Disease Programs, Bayer AG Pharmaceuticals, Berlin, Germany; Sustainability, Eisai Co. Ltd, Tokyo, Japan; Novartis Global Health & Corporate Responsibility, Basel, Switzerland; Department of Control of Neglected Tropical Diseases, WHO Geneva, Geneva, Switzerland; Department of Control of Neglected Tropical Diseases, WHO Geneva, Geneva, Switzerland; Department of Communicable Diseases WHO Regional Office for South-East Asia, Delhi, India; Expanded Special Project for Elimination of NTDs (ESPEN) WHO Regional Office for Africa, Brazzaville, Congo; Division of Programs for Disease Control WHO Regional Office for the Western Pacific, Manila, Philippines; NTD Support Centre, Task Force for Global Health Atlanta, USA

**Keywords:** endemic countries, medicine donations, neglected tropical diseases, partnerships, pharmaceutical companies, 2030 NTD roadmap

## Abstract

Neglected tropical diseases (NTDs) are targeted for global control or elimination. Recognising that the populations most in need of medicines to target NTDs are those least able to support and sustain them financially, the pharmaceutical industry created mechanisms for donating medicines and expertise to affected countries through partnerships with the WHO, development agencies, non-governmental organisations and philanthropic donors. In the last 30 y, companies have established programmes to donate 17 different medicines to overcome the burden of NTDs. Billions of tablets, capsules, intravenous and oral solutions have been donated, along with the manufacturing, supply chains and research necessary to support these efforts. Industry engagement has stimulated other donors to support NTDs with funds and oversight so that the ‘heath benefit’ return on investment in these programmes is truly a ‘best value in public health’. Many current donations are ‘open-ended’, promising support as long as necessary to achieve defined health targets. Extraordinary global health advances have been made in filariasis, onchocerciasis, trachoma, trypanosomiasis, leishmaniasis, schistosomiasis, intestinal parasites and others; and these advances are taking place in the context of strengthening health systems and meeting the global development goals espoused by the WHO. The pharmaceutical manufacturers, already strong collaborators in initiating or supporting these disease-targeted programmes, have committed to continuing their partnership roles in striving to meet the targets of the WHO's new NTD roadmap to 2030.

## Medicine donation programmes for the neglected tropical diseases

Among the ‘diseases of poverty’ is a group of infectious diseases, the neglected tropical diseases (NTDs), which affect literally hundreds of millions of people, often in the world's most underserved populations.^1^ Until recently, these diseases were particularly troubling to the global health community because while the medicines needed to control or even eliminate them already existed, they were not generally available or accessible to those who needed them.

Bridging this divide between availability and need had long been the province of philanthropic and development organisations, but starting in the 1980s a significant transformation began when the manufacturers of the needed pharmaceuticals created a new strategy based on large-scale donations to make medicines for many of the NTDs available at no cost to national health programmes and their populations in need, all while partnering with the WHO, non-governmental organisations (NGOs), development agencies and the ministries of health in NTD-endemic countries.^1^

It was Merck & Co. Inc., Rahway, NJ, USA in 1987 that pioneered this NTD drug-donation model with a pledge to provide its medicine Mectizan (ivermectin) to all who need it for as long as required to control river blindness (onchocerciasis) globally.^2^ A key rationale for this decision to donate the medicine was the recognition that those who needed the medicine most would not be able to afford it at any price. This decision soon stimulated other pharmaceutical companies to make similar donation commitments that have now enabled the treatment of additional NTDs as well (see below).

In 2012, a broadly representative meeting of partners was held in London to accelerate progress toward the control and elimination of NTDs. Specific pledges were announced (codified in a London Declaration^3^) by pharmaceutical companies, bilateral development agencies, NGOs and other partners to support the WHO's 2020 roadmap for NTDs, which served as a guide to accelerate programme progress. Within 5 y of the London Declaration, the volume of donated medicines delivered to NTD programmes had more than doubled, and by 2020 17 different drug products had been donated (Table 1). These medicine donations, accompanied by additional contributions from industry, have proven to be critically important in supporting NTD programme activity both by offsetting costs to national programmes and by stimulating increased collaborative support from bilateral development agencies (especially from US, UK and Japanese governments) and from private philanthropy. Collectively, these agencies and organisations have recognised the unique value that the medicine donations bring to ever-expanding global health partnerships; indeed, the investments to programmes targeting the NTDs have been calculated as offering a net benefit to affected populations of almost US$25 for every dollar invested, equating to a 30% annualised rate of return.^4^ For this reason—and the substantial health impact achieved—these donation-based NTD programmes are now identified as truly a ‘best value in public health’.^5-7^

**Table 1.** Essential NTD medicines donated by pharmaceutical companies through the WHO and other agencies (data sourced from the WHO's 2030 NTD draft roadmap^22^ and independent industry consultation)

Company	Medicine	Commitment	Donation
Merck & Co., Inc., Rahway, NJ, USA	ivermectin (3 mg tablets)	Since 1987 until the global elimination of ONCHOSince 1997 until the elimination of LF in Yemen and African countries, where LF and ONCHO are co- endemic2018–2025 for use in the WHO-recommended triple-therapy MDA regimen to eliminate LF in countries not co-endemic for ONCHO	Unlimited supplyUnlimited supplyUp to 100 million treatments(9 .6 billion tablets, equalling more than 3.4 billion treatments, donated during the last 29 y; 2019 annual donation was >962 million tablets, equalling >340 million treatments)Donated through the MDP
GlaxoSmithKline	albendazole (400 mg tablets)	Since 1997 until global elimination of LF as a public health problem is achieved2012–2020 for use in the PC of STH in SAC	Up to 600 million tablets annuallyDonation expanded by an additional 400 million tablets annually9.8 billion tablets donated during the last 20 yDonated through the WHO
Pfizer	azithromycin (250 mg tablets + Paediatric Oral Suspension)	Since 1998–2020 for the elimination of trachoma as a public health problemCommitment extended to 2025	Unlimited quantity900 million doses donated to dateDonated through the ITI
Novartis	Multi-drug therapy (rifampicin,clofazimine, dapsone)Clofazimine capsulesTriclabendazolecapsules	Since 2000–2020 for the treatment of leprosy and its complications2000–2020 for the treatment of severe erythema nodosum leprosum reactions2005–2022 for the treatment of fascioliasis and paragonimiasis	Unlimited supplySince 2000, donation has reached >7 million patientsUnlimited supplyLatest worldwide donation through the WHO (January 2019 to December 2022) includes the donation of 600 000 annually, expected to reach 300 000 patients per year. Since 2005, the donation amounts to approximately 4 million tablets
Sanofi	eflornithine (100 mg/kg)	Since 2001–2020 for the treatment of HAT	Unlimited quantityDonated through the WHO
	melarsoprol (3.6 mg/kg/day)	Since 2001–2020 for the treatment of HAT	Unlimited quantityDonated through the WHO
	pentamidine (300 mg i.v. solution)fexinidazole (600 mg tablets)	Since 2001–2020 for the treatment of HATSince 2020 for the treatment of HAT	Unlimited quantityFor as long as neededDonated through the WHO
Bayer	Suramin (1 g vials for solution)	Since 2002–2020 Donated quantities according to the WHO request for treatment of HAT *rhodesiense*	Donated through the WHOBased on 5-y supply agreements with the WHO with annual review of demand
	nifurtimox (120 mg tablets)	2019–2021 Starting with the WHO launch of the NECT, donated quantities according to the WHO request for the treatment of HAT *gambiense*Partnership with Sanofi (eflornithine)	
	nifurtimox (120 mg tablets)	2004–2021 Donated quantities according to the WHO request for the treatment of Chagas disease (American trypanosomiasis)	
	niclosamide (400 mg tablets)	2020–2024 Donated quantities according to the WHO request for treatment of taeniasis/cysticercosis	
	praziquantel (600 mg tablets)	2020–2024 Donated quantities according to the WHO request for treatment of taeniasis/cysticercosis	
Johnson & Johnson	mebendazole (500 mg tablets)	Since 2006–2020 for the treatment of STH in SAC2010 revised volume commitment2019 revised duration of commitment through 2025	Initially 50 million treatments annually2010 revised to 200 million treatments annually2019 extended 200 million annual donationDonated through the WHO
Merck KGaA, Darmstadt, Germany	praziquantel (600 mg tablets)	2007–2017 (initial 10-y period) to treat SCH in SAC	Up to 200 million tablets over 10 y
		2017 revised duration commitment for an unlimited perioduntil elimination of schistosomiasis as a public health problem	Donation scaled up to 250 million tablets annuallyMore than 1 billion tablets donated to dateDonated through the WHO
Gilead	liposomal amphotericin B (lyophilised 50 mg formulation)	Since 2011–2016 for the treatment of VL in South-East Asia and East Africa2017–2021 revised duration commitment for the treatment of VL in South-East Asia and East Africa	Up to 450 000 vials to treat 50 000 people over 5 yUp to 380 000 additional vialsDonated through the WHO
Eisai	DEC (100 mg tablets)	Since 2013–2020 for use in the PC of LF2017 revised duration commitment until elimination of LF as a public health problem achieved	Up to 2.2 billion tablets committed for first 7-y periodExtended until elimination is achievedDonated through the WHO
EMS SA Pharma (Brazil)	azithromycin (500 mg tablets)	2019–2023 to support the global eradication of yaws.	153 million tabletsDonated through the WHO

Abbreviations: DEC, diethylcarbamazine citrate; HAT, human African trypanosomiasis; ITI, International Trachoma Initiative; i.v., intravenous; LF, lymphatic filariasis; MDA, mass drug administration; MDP, Mectizan Donation Program; NECT, nifurtimox eflornithine combination treatment; ONCHO, onchocerciasis; PC, preventive chemotherapy; SAC, school-aged children; SCH, schistosomiasis; STH, soil-transmitted helminthiasis; VL, visceral leishmaniasis.

## Current industry commitments

Table 1 identifies the pharmaceutical industry's major partnership commitments to NTD programmes over the last 33 y. It includes a list of companies involved, medicines donated and the duration of company commitments to support the NTD programmes, as well as the most recent revisions and extensions of these commitments. Interestingly, too, it also depicts how industry has responded to evolving NTD programmatic needs and to the expansion of the NTD focus over time.

It has become clear that industry contributions of medicines are critically important for the success of today's NTD programmes, with their fair market value exceeding several hundred million dollars annually. Table 1 does not, however, capture the additional financial, human resource and other contributions provided by the pharmaceutical companies or the contributions by member state governments, bilateral agencies, NGOs and other private sector donors, although these additional inputs are considerable and, of course, essential for programme success.

**Figure 1.**
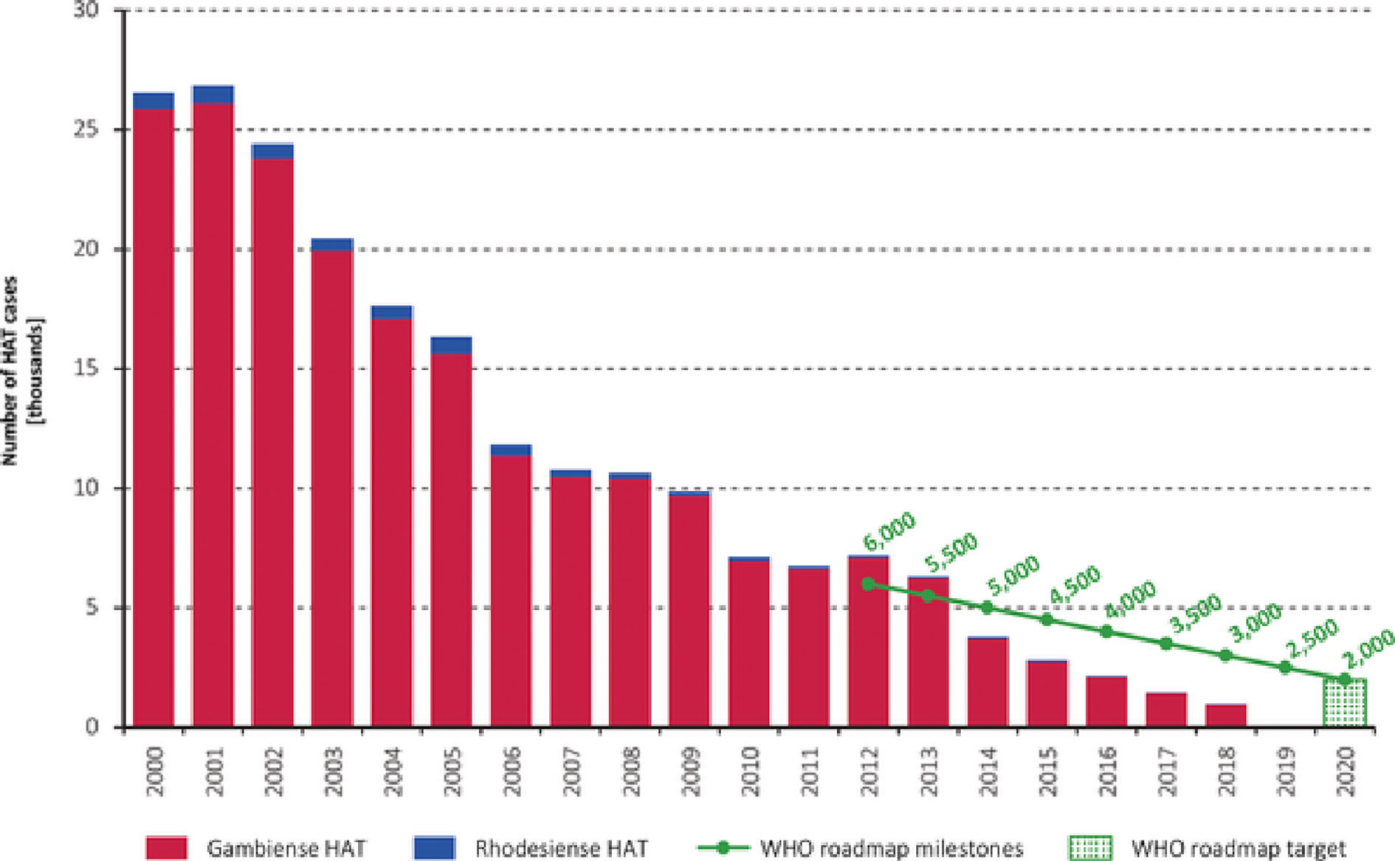
Total number of reported cases of human African trypanosomiasis (HAT) (*gambiense* and *rhodesiense*) per year (2000–2018).^8^

**Figure 2.**
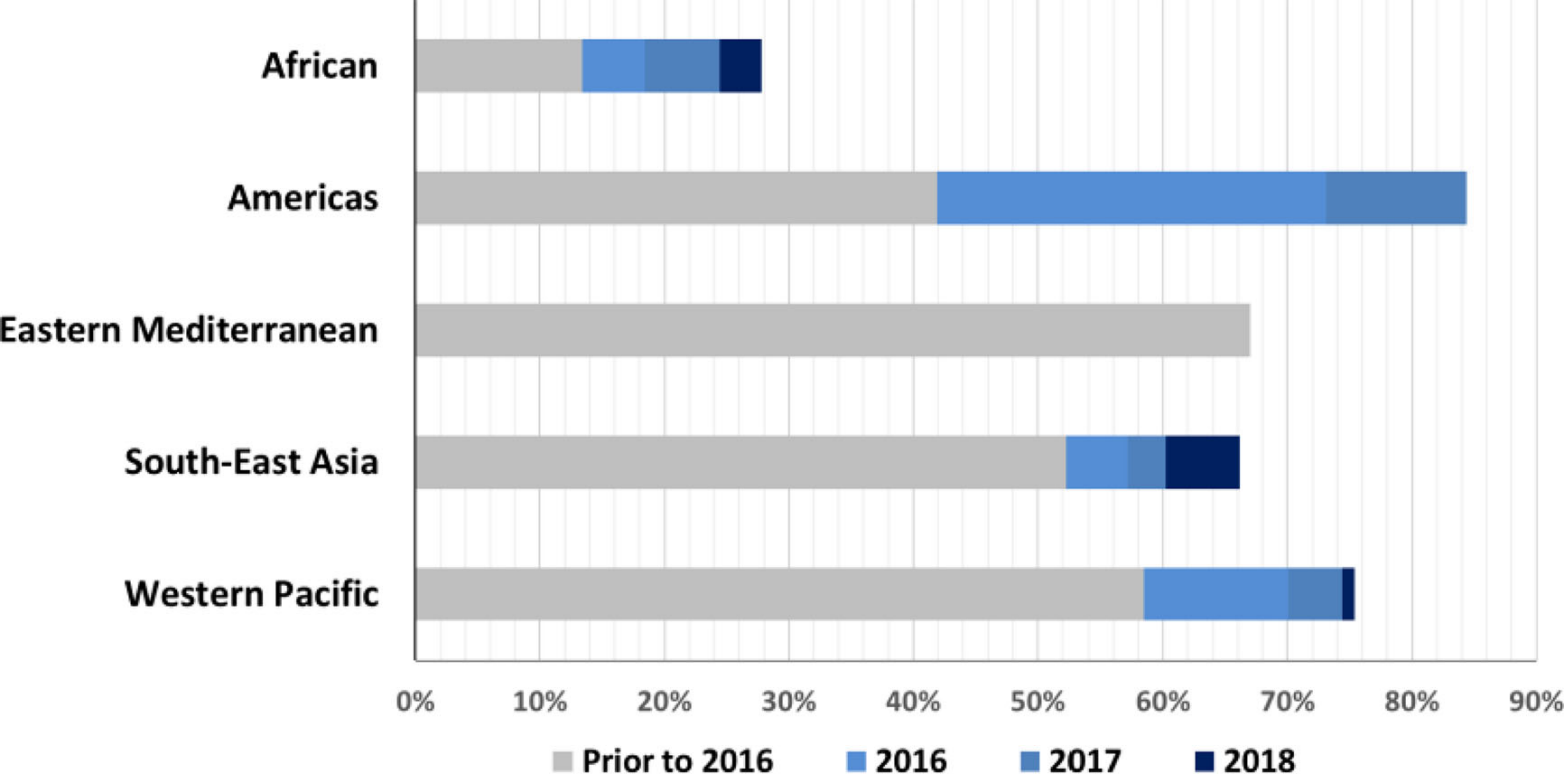
Proportion of implementation units that have completed transmission assessment surveys (TAS) for lymphatic filariasis (LF) and no longer require mass drug administration (MDA)^9^ (by WHO region).

**Figure 3.**
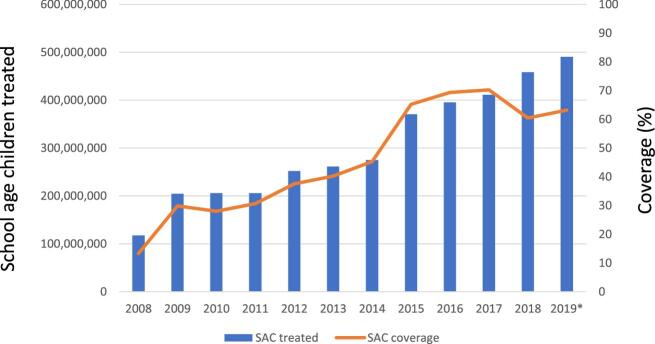
The number of school-aged children treated for soil-transmitted helminthiases (STH) 2008–2109.^10^

**Figure 4.**
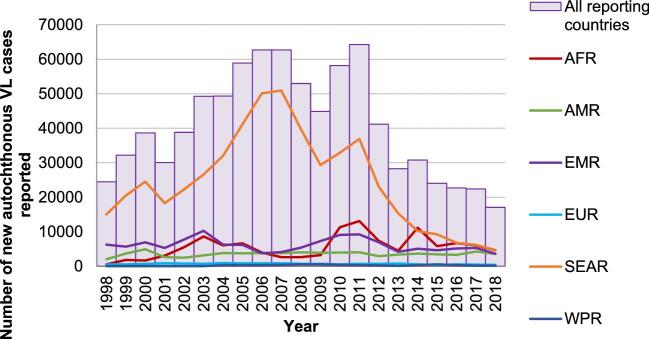
Evolution of numbers of visceral leishmaniasis (VL) cases, by WHO region, 1998–2018.^11^

When public health is assessed with the same rigour as other businesses, a principal focus must be on the added value derived from specific investments. Certainly, the medicine donations are not responsible for all the benefits derived from NTD programmes, but Table 2 highlights some of the remarkable achievements these programmes have made while they have been supported by the donated medicines. Similarly, Figures 1–4 also identify the dramatic changes observed over time for a selection of NTDs whose performance indicators illustrate the important public health impact that has come from these donation-facilitated NTD programmes, that is, the declining numbers of human African trypanosomiasis (HAT) cases,^8^ the formerly endemic areas freed from lymphatic filariasis,^9^ the increases in the numbers of school children treated each year for intestinal worms^10^ and the progressively decreasing numbers of visceral leishmaniasis cases over time.^11^ The medicine donation programmes are not solely responsible for these programmatic achievements, but without them (and the collaborative support that they attract) there would likely be much less impact of any of the initiatives targeting these NTDs.

**Table 2.** Reach and impact of the donation-backed programmes targeting NTDs

Diseases	Results and milestones
HAT	Since 2001 more than 40 million people have been screened and >210 000 people diagnosed and treated^23^Since 2001 the number of cases has decreased by 96% (Figure 1)In 2019, just 997 cases of *T. gambiense* were reported compared with nearly 10 000 cases in 2009^8^In 2020, the elimination of HAT *gambiense* as a public health problem was declared by the WHO
Trachoma	As of April 2019, 9 countries have been validated as achieving trachoma elimination goalsThe number of people at risk of trachoma has fallen from 1.5 billion in 2002 to around 142 million in 2019, a decrease of 91%The number of people requiring surgery for trachomatous trichiasis decreased by 74% from 2002 to 2019^26^
ONCHO	The disease has been eliminated in Colombia, Ecuador, Guatemala and MexicoEliminated in some foci in Ethiopia, Mali, Nigeria, Sudan, Uganda and VenezuelaIvermectin treatment has scaled to reach 83.9% geographic coverage of known endemic areasOver 6.5 million people no longer require treatment as areas are under post-treatment surveillance^27^
LF	17 countries have been validated as having eliminated LF as a public health problemOver 550 million people no longer require PC, a reduction of 42% in the at-risk population since 2010^9^ (Figure 2)From 2000 to 2018, there has been an estimated 74% reduction in the number of infected people^28^
STH (intestinal worms)	Over 500 000 DALYs averted in 2015 (44% of the annual burden in children); the number of the DALYs averted is expected to be 900 000 in 2020^29^In 2018, 576 million children received treatment for STH covering 54% of children in need globally^10^ (Figure 3)From 2008 and 2019, 3.6 billion SAC received treatment^29^
SCH	From 2008–2019, treatment of 400 million SAC in AfricaTreatment of 72% of children endangered by the disease in sub-Saharan Africa in 2017^10^
VL	Since 2011, the number of VL cases has decreased consistently to the current level of 17 082 cases, which is the lowest number since 1998 (Figure 4)In South East Asia, VL morbidity was reduced by over 82% and the case fatality rate has decreased by 95%However, the number of new autochthonous cases of cutaneous leishmaniasis reported to the WHO from 1998 to 2018 increased from 71 486 to 251 553^11^
Chagas disease/American Trypanosomiasis	Between 1980 and 2010the number of infected people declined from 17.4 million to 5.7 millionThe number of new cases per year declined from 700 000 to 38 593The number of deaths per year declined from >45 000 to 12 000^30,31^
Leprosy	For the last decade, >200 000 people have been newly diagnosed with leprosy every year, including thousands of children^32^
Fascioliasis	Since 2005, 2 million people with fascioliasis have been treated in >30 countries worldwide^33^

Abbreviations: DALYs, disability-adjusted life years; HAT, human African trypanosomiasis; LF, lymphatic filariasis; ONCHO, onchocerciasis; PC, preventive chemotherapy; SAC, school-aged children; SCH, schistosomiasis; STH, soil-transmitted helminthiasis; VL, visceral leishmaniasis.

## Lessons learned from the medicine donation programmes

While the operational complexities that pharmaceutical companies experience in implementing their donation programmes can vary as much as the disease characteristics and treatment protocols of the individual NTDs they support, there are common learned lessons that are important to note, in particular the value of partnerships, supply chain information sharing and operational research.

As the first company to establish a large-scale NTD donation programme, Merck & Co., Inc., Rahway, NJ, USA learned many lessons regarding the distribution of Mectizan that were shared and subsequently incorporated in later donation programmes.^12^ Foremost among these lessons was the importance of the very partnerships themselves.^13^ Through collaboration, each partner (including the WHO, the pharmaceutical companies, the development agencies and the NGOs) could bring its unique comparative advantage to benefit national NTD programmes and the communities they serve. Indeed, partnerships between competing companies who routinely rivalled each other commercially enabled these companies at the same time to cooperate closely in their philanthropic efforts.^14^ These collaborations have expanded and evolved with time to meet the needs—both operational and political—of an ever-increasing number of medicine donation programmes that now include, importantly, both those of companies with a focus on diagnosing and treating conditions affecting entire communities^15^ and those with a focus on treating diseases of individual patients.^8^

Partnership has also proven to be key to addressing important challenges associated with manufacturing and supply chain management for NTD medicines. Donation programmes often pose greater challenges than the manufacturing and distribution of commercial products because demand forecasting at country level is not always accurate and often is misaligned with manufacturing capacity timelines. Shipping product into resource-poor countries can also be fraught with delays and unanticipated issues. To address some of these challenges, the NTD Supply Chain Forum was established as a public-private partnership in 2012 to serve as a common platform for engagement of supply chain experts from the WHO, pharmaceutical companies, NGOs, ministries of health and logistics providers.^16^ The Forum created NTDeliver as a centralised information system for storing annual requests and shipping information related to donations in an effort to mitigate these issues. Challenges still exist; for example, as a result of the severe acute respiratory syndrome coronavirus 2 pandemic, demand forecasting and supply chain logistics have become even more difficult in 2020, with major disruptions to planned NTD activities. Clearly, from end to end, there remains the need for more timely, better quality data to support supply chain management, but the NTD Supply Chain Forum has proven to be a great advantage in navigating these ongoing challenges.

An additional operational challenge for the donation programmes has been the adjustment required to support updated or new disease targets and treatment guidelines: necessary either because the strategy in an existing global programme has changed or because a wholly new disease challenge has been targeted. While perhaps appearing as a simple extension of the earlier programmes, such new or revised initiatives require the same (often lengthy and expensive) preliminary and pilot studies required of the earlier programmes. Global guidance and stakeholder alignment are necessary to effectively support these transitions and new programmes, in order for the pharmaceutical partners to be efficient and maximise the benefits that their donations can provide. A robust operational research community has been critical to providing the sound empirical evidence companies need to make these decisions.^17^

## Innovation of new products: research and development

While existing donations form a necessary and significant component of the global response to NTDs, research and development (R&D) into innovative new products—whether in the form of new drugs, diagnostics or vaccines—is still essential to achieving long-term, sustained NTD elimination or control. Pharmaceutical companies are already actively engaged in R&D for NTDs; for example, in formulating paediatric-friendly dosing options for both schistosomiasis (Merck KGaA and Astellas) and soil-transmitted helminths (Johnson & Johnson). However, maintaining the required level of investment needed to bring new treatments for NTDs to market remains challenging. The NTDs tend to predominate in settings where economic stresses often limit options for large-scale public-sector procurement and individual patient access to new medical interventions. Consequently, opportunities to recover the costs associated with the R&D of new therapeutics are limited. Despite these constraints, industry has continued to invest in innovation for NTDs facilitated by sharing the risk throughout the product's development lifecycle and leveraging the expertise of various partners involved in delivering healthcare. Several mechanisms are available to facilitate product development partnerships, including the Drugs for Neglected Diseases initiative (DNDi), the Global Health Innovative Technology Fund, GSK's Open Lab Foundation, the World Intellectual Property Organisation (WIPO) and Bio Ventures for Global Health.^18^ Such partnerships can be extremely successful, as, for example, in the partnership between Sanofi and DNDi, which culminated in the development of fexinidazole, an innovative solution for HAT that allows oral treatment for both stages of the disease, avoiding the need for risky and invasive lumbar puncture and hospitalisation.^19^ This €68 million project was supported by seven European countries, the Bill & Melinda Gates Foundation and Médecins Sans Frontières. Sanofi has pledged to donate the drug free of charge for as long as it is needed.

Ultimately, there is no single solution to R&D challenges and as we move forward, a combination of several incentive models that could sustain and further stimulate R&D for NTDs will need to be considered,^20^ starting with existing mechanisms for stimulating R&D that include the WIPO initiative for global dissemination of intellectual property data, advance market commitments, voluntary patent pools, priority review vouchers, increased public funding and/or collaboration for high risk phase III clinical trials, streamlined regulatory processes and increased funding for the scaling up of production facilities. None of these mechanisms is a standalone solution and future efforts will likely succeed only if they supplement, rather than try to replace, existing market-based incentives.

## Donation commitments beyond 2020: industry support for the new 2021–2030 NTD roadmap

Drug donations have played an important role in supporting WHO aspirations across a broad range of NTD-focused global health initiatives.^21^ Over the last 3 decades NTDs have attracted increased attention and investment, and with only a few exceptions the availability of drugs is no longer viewed as a significant barrier to achieving both programme targets and the objectives outlined in the Sustainable Development Goals (SDGs) and Universal Health Coverage (UHC). Indeed, substantial progress has been made towards achieving WHO targets set in 2012.^22,23^ For example, over 500 million fewer people require interventions against NTDs than in 2010, and 40 countries, territories and local areas have eliminated at least one disease. Lymphatic filariasis and trachoma have been eliminated as a public health problem in 17 and 9 countries respectively, and reported cases of HAT have fallen dramatically over the last 20 y.

Driven by a recommendation from the 146th WHO Executive Board meeting in 2020, the WHO has developed, through a broadly consultative process, a revised NTD roadmap and an accompanying sustainability framework for action to address the needs and define strategies to support successful NTD programme delivery over the next decade.^22^ The roadmap

- sets aspirational disease-specific targets for 20 public health concerns; addresses strengthening the capacity of national health systems to facilitate programme delivery through existing health and education infrastructure and an invigorated multisectoral engagement of state and non-state actors responsible for delivering vector control, water security, sanitation, animal welfare and environmental health services;
- aims to improve the sustainability and efficiency of national NTD programmes to ensure that all patients have equitable access to treatment, care and support to ensure that the gains from NTD control and elimination efforts are effectively translated into long-term human development gains;
- underscores the notion that as drivers and beneficiaries of this broad global health agenda, member state government agencies are expected to define and deliver multisectoral national plans for NTDs that are domestically financed, informed by quality data and fully integrated into the governance and administration of health, education and other public service delivery systems.

Accelerating the transition from a donor-dependent model to one that is focused on domestic resource mobilisation will strengthen country ownership and contribute significantly towards the achievement of UHC. Collectively, by investing in NTD elimination and control programmes, national governments and private and bilateral stakeholders can transform the socioeconomic prospects of affected communities and countries worldwide. Achieving the goals identified in the NTD roadmap will require long-term, dependable access to quality-assured NTD medicines, diagnostics and other health products. To meet these challenges, industry partners are committed to the long-term donation of quality-assured medicines, innovative R&D, financial support and advocacy in support of the global health agenda targeting the NTDs,^18,24^ including:

1. the elimination targets for onchocerciasis, lymphatic filariasis, trachoma and HAT, as well as the focal elimination targets of schistosomiasis;
2. the control of visceral and cutaneous leishmaniasis, Chagas disease, soil transmitted helminths and some food-borne trematodes;
3. innovative means to accelerate global progress against NTDs, ensuring that activities are sustainable, have a real impact and are increasingly owned and directed by member states;
4. acceleration of R&D, including through innovative public-private mechanisms to identify and develop new drugs, vaccines and diagnostics necessary to ensure long-term sustainable control and elimination of NTDs;
5. collaboration to strengthen supply chain operations, from the first to the last mile;
6. advocacy to raise awareness about the resources needed to remove the two primary risk factors for NTDs—poverty and exposure to disease—along with ensuring access to clean water and basic sanitation, improving living conditions, providing vector control, health and education, and strengthening health systems in endemic areas, all of which are essential for NTD elimination.

Industry partners continue to prioritise NTD investments because they have determined that the commitment of these resources is well worth the resultant public health impact. However, making the case for continued investments requires demonstrated measurable progress towards each programme's public health objectives. Strong national programmes that align with global targets and include rigorous monitoring and evaluation indicators, along with the necessary domestic and external support for delivering the medicines, are critical for providing a rationale for sustained company investment. The WHO 2030 roadmap^22^ provides a framework to guide national NTD programmes and allow industry partners to measure progress towards the targets their investments support.

### Conclusions

2020 is a landmark year for NTDs. The London Declaration—a collective commitment to bring resources to bear to end NTDs—was launched in 2012 in support of the WHO's NTD goals until 2020.

A new NTD era (until 2030) is now being forged by the WHO with its international partners, including the pharmaceutical companies. This year also marks the 10-y countdown to the achievement of the SDGs, which includes SDG target 3.3 to ‘End the epidemics of AIDS, TB, malaria, NTDs and other communicable diseases’.

Over the last 3 decades industry has demonstrated, through the donation of disease-targeted therapeutics, a willingness to engage with member states, the WHO and other partners to address the burden of NTDs and contribute to their control and elimination. Despite the fact that decades-long programmes are an unusual phenomenon in the pharmaceutical industry, NTD commitments are stronger today than at any time in the past. Long-term product commitments to achieve elimination goals or to provide unlimited quantities of medicines to achieve control targets have been pledged for at least 9 of the 17 products currently donated. The political will to address NTDs was exemplified in 2019 at the United Nations High Level Meeting on UHC,^25^ which recognised that access to NTD interventions is integral to achieving ‘Health for All’.

Today, medicine donations are a key component of the innovative pharmaceutical industry's support to address NTDs. These programmes have become the industry's gateway to multidimensional activities focused not only on the provision of medicines but also on health education, R&D activities and health system strengthening, sharing with other global partners the common goal of a world free of NTDs. Industry, with its particular competencies, experience and resources, has indeed become a valued, reliable and trusted partner in addressing the NTD health needs of underserved populations globally.

## Data Availability

None.
