# Corrigendum to: Medicine donation programmes supporting the global drive to end the burden of neglected tropical diseases

**DOI:** 10.1093/trstmh/trab046

**Published:** 2021-03-16

**Authors:** Mark Bradley, Rachel Taylor, Julie Jacobson, Morgane Guex, Adrian Hopkins, Julie Jensen, Lynn Leonard, Johannes Waltz, Luc Kuykens, Papa Salif Sow, Ulrich-Dietmar Madeja, Takayuki Hidal, Kileken Ole-Moiyoi, Jonathan King, Daniel Argaw, Jamsheed Mohamed, Maria Rebollo Polo, Aya Yajima, Eric Ottesen

**Affiliations:** Global Health, GSK, Brentford, UK; Corporate Responsibility, Merck & Co., Inc., Kenilworth, NJ, USA; Bridges to Development, Seattle, Washington, USA; International Federation of Pharmaceutical Manufacturers and Associations, Geneva, Switzerland; Adrian Hopkins Consulting, Gravesend, Kent, UK; Global Health & Patient Access, Pfizer, Inc. New York, NY, USA; Johnson & Johnson Global Public Health, New Brunswick, NJ, USA; Global Health, Group Corporate Affairs Merck KGaA, Darmstadt, Germany; Global Health Programs, Sanofi, Paris, France; Global Patient Solutions Africa, Gilead Sciences, Foster City, CA, USA; Neglected Tropical Disease Programs, Bayer AG Pharmaceuticals, Berlin, Germany; Sustainability, Eisai Co. Ltd, Tokyo, Japan; Novartis Global Health & Corporate Responsibility, Basel, Switzerland; Department of Control of Neglected Tropical Diseases, WHO Geneva, Geneva, Switzerland; Department of Control of Neglected Tropical Diseases, WHO Geneva, Geneva, Switzerland; Department of Communicable Diseases WHO Regional Office for South-East Asia, Delhi, India; Expanded Special Project for Elimination of NTDs (ESPEN) WHO Regional Office for Africa, Brazzaville, Congo; Division of Programs for Disease Control WHO Regional Office for the Western Pacific, Manila, Philippines; NTD Support Centre, Task Force for Global Health Atlanta, USA

Transactions of the Royal Society of Tropical Medicine and Hygiene, traa167, https://doi.org/10.1093/trstmh/traa167

In the originally published version of this article, errors relating to the incorrect use of the Merck corporate name were identified. This corrigendum addresses the changes that have subsequently been made in the ‘Medicine donation programmes for the neglected tropical diseases’ and ‘Lessons learned from the medicine donation Programmes’ sections. In both sections, mention of 'Merck & Co., Inc' should read: ‘Merck & Co., Inc., Rahway, NJ, USA’.

